# Depression in the Iranian Elderly: A Systematic Review and Meta-Analysis

**DOI:** 10.1155/2021/9305624

**Published:** 2021-08-16

**Authors:** Hedayat Jafari, Dariush Ghasemi-Semeskandeh, Amir Hossein Goudarzian, Tahereh Heidari, Azar Jafari-Koulaee

**Affiliations:** ^1^Nursing Department of Medical Surgical Nursing, Traditional and Complementary Medicine Research Center, Addiction Institute, Mazandaran University of Medical Sciences, Sari, Iran; ^2^Institute for Biomedicine, Eurac Research Institute, Bolzano, Italy; ^3^Department of Genetics, Leiden University Medical Center, Leiden, Netherlands; ^4^Research Committee, Faculty of Health, Mazandaran University of Medical Sciences, Sari, Iran; ^5^Student Research Committee, Mazandaran University of Medical Sciences, Sari, Iran

## Abstract

Depression can lead to increased medical costs, impaired individual and social functioning, nonadherence to therapeutic proceeding, and even suicide and ultimately affect quality of life. It is important to know the extent of its prevalence for successful planning in this regard. This study was conducted to determine the prevalence of depression in the Iranian elderly. This systematic review and meta-analysis study was done through Medline via PubMed, SCOPUS, Web of Science, ProQuest, SID, Embase, and Magiran with determined keywords. Screening was done on the basis of relevance to the purpose of the study, titles, abstracts, full text, and inclusion and exclusion criteria. The quality of the articles was assessed using the Newcastle-Ottawa standard scale. After primary and secondary screening, 30 articles were finally included in the study. According to the 30 articles reviewed, the prevalence of depression in the Iranian elderly was 52 percent based on the random-effects model (CI 95%: 46–58). According to the results of the present study, depression in the Iranian elderly was moderate to high. Therefore, more exact assessment in terms of depression screening in elderly people seems necessary. Coherent and systematic programs, including psychosocial empowerment counselling for the elderly and workshops for their families, are also needed. Researchers can also use the results of this study for future research.

## 1. Introduction

The phenomenon of aging as one of the most sensitive periods of human life is one of the most important economic, social, and health challenges in the world that is expanding with the growing trend [1, 2]. Statistics show that the world population over 60 will double between 2000 and 2050, reaching from 11 to 22 percent [3, 4]. The age pyramid of the Iranian population is also moving from youth to old age, so that the number of elderly people in 1414 reached more than 10 million and the percentage of aging reached more than 11% [5]. The aging process and its associated changes may make the elderly more vulnerable to potential threats such as chronic illnesses [6, 7], loneliness and isolation [8], anxiety, and depression [9]. Mental health disorders have also been reported as the most common disorders in the elderly, with depression being the most prevalent [10, 11]. It was reported that approximately 5 million older adults worldwide experience late-onset depression [12]. According to the World Health Organization, depression is characterized by persistent sadness and lack of interest or enjoyment previously or in pleasurable activities [13]. Unfortunately, depression is less common in the elderly. Aging depression is a condition that does not occur naturally due to aging, and the general symptoms that are a prominent symptom of depression may not be seen. Most of the depressed elderly have fewer complaints of feelings of sadness and grief caused by a depressed mood. Mental preoccupation with health status and extreme and obsessive attention to physical symptoms are other characteristics of depression in the elderly. Another common symptom of depression in the elderly is a complaint of cognitive symptoms such as forgetfulness and distraction, so that the term false dementia is used to describe this clinical picture [14–16]. Factors such as loneliness, decreased ability to work, impaired social support, having one or more physical ailments, and multiple therapeutic interventions can be risk factors for depression in the elderly [17]. Depression in the elderly can lead to increased medical costs, impaired individual, occupational and social functioning, nonadherence to therapeutic proceeding, and even suicide and ultimately affect one's quality of life [18, 19]. Depression is also one of the most important predictors of treatment outcome and survival in these patients [20]. Therefore, it is important to pay attention to the challenges and psychological needs of the elderly as well as identify and evaluate the prevalence of depression in order to prevent depression and its unpleasant side effects and to achieve successful aging and optimal quality of life. A review of the literature revealed that there have been several descriptive studies on the prevalence of depression in the Iranian elderly [21, 22], and so far, only one review study has been conducted in Iran that has reviewed articles on depression in the Iranian elderly by 2015 [23]. Given the time elapsed from this study, it is necessary to conduct more comprehensive study with more accurate and generalizable methods for continuous evaluation. Therefore, the present study was conducted to determine depression in the Iranian elderly in a systematic review and meta-analysis.

## 2. Materials and Methods

This systematic review and meta-analysis was performed based on Preferred Reporting Items for Systematic Reviews and Meta-Analyses (PRISMA) with the aim of determining depression in the Iranian elderly in 2020.

### 2.1. Search Strategy

We searched Medline via PubMed, SCOPUS, Web of Science, ProQuest, SID, EMBASE, and Magiran, from the earliest date possible until the end of 2019. Databases were searched by using keywords including “Depression” OR “Depressive Disorders,” “Iran,” “Prevalence,” and “Elderly OR Aged OR Older adult.” Boolean operators (AND and OR) were used. After searching the database, a list of articles was prepared by two researchers independently and the duplicate articles were removed. The studies were screened based on the titles, abstracts, and full text of the articles. Finally, eligible articles were included in the study process based on inclusion and exclusion criteria.

### 2.2. Inclusion and Exclusion Criteria

The inclusion criteria were the following: (1) observational studies with keywords listed in the title or abstract of the articles; (2) published in Persian or English; (3) studies with samples of Iranian nationality residing in Iran. Exclusion criteria included the following: (1) low-quality studies based on scale; (2) no relation with the subject and inadequate data such as failure to report the percentage of depression. Also, books and studies without full text and studies in format of abstracts of conference papers were excluded.

### 2.3. Quality Assessment of Studies

The quality of the selected articles was assessed based on the Newcastle-Ottawa scale. This scale measures the research design, response rate, sample representation, objectivity/reliability of the result, computation power provided, and appropriate statistical analysis. Finally, articles with a score of at least 7 were included in the study. The quality of studies was evaluated independently by two researchers, and the third researcher was used in case of disagreement.

### 2.4. Data Extraction

Data were extracted using a checklist consisting of first author's name, year of publication, type of study, place of study, sample size, mean age of participants, and percentage of depression. The necessary information was extracted from related articles by two independent researchers.

### 2.5. Statistical Analysis

The studies reporting the prevalence of depression in the Iranian elderly samples were synthesized using both fixed-effects and random-effects models. The method to be used for pooling of studies was inverse variance. Statistical heterogeneity among reviewed studies was also assessed with *I*2 statistic. Potential publication bias was visually evaluated with a funnel plot and statistically tested by Egger's test statistic which is based on a weighted linear regression of the treatment effect on its standard error [24]. Statistical analyses were implemented on *R* version 4.1.0 [25] via metafor package [26].

## 3. Results

**Figure 1:**
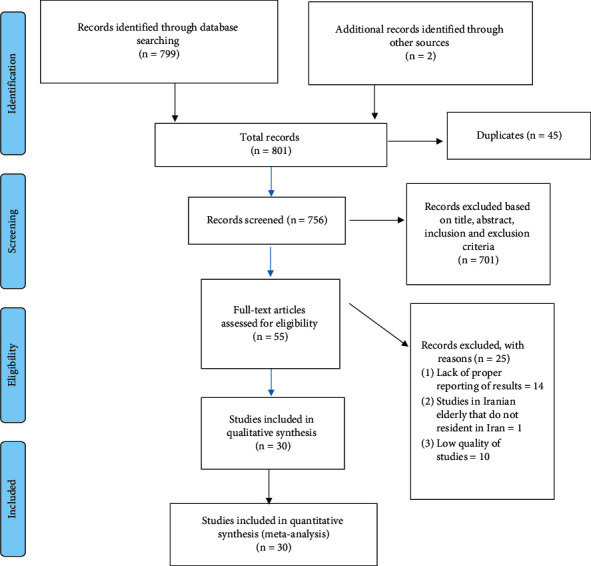
Process of study selection (PRISMA).

After primary and secondary screening, 30 studies were finally included in the systematic review and meta-analysis. Details of screening steps are presented in Figure 1.

### 3.1. Description of Studies

Thirty articles included in this study were all observational studies and had a good locative distribution throughout Iran. GDS, BDI, Beck, DASS-21, Kessler, HADS, and Elderly Health Master Plan tools were used to assess depression in the elderly. The age range of the participants ranged from 64.22 to 76.97. Other details are presented in Table 1.

### 3.2. Meta-Analysis Results

The range of sample size from the included articles varied from 52 to 9965, with a mean and median of 863 and 315, respectively. Based on the 30 identified articles, the reported prevalence of depression ranged from 9 to 100 percent, with the average of 52.15 percent.

**Figure 2:**
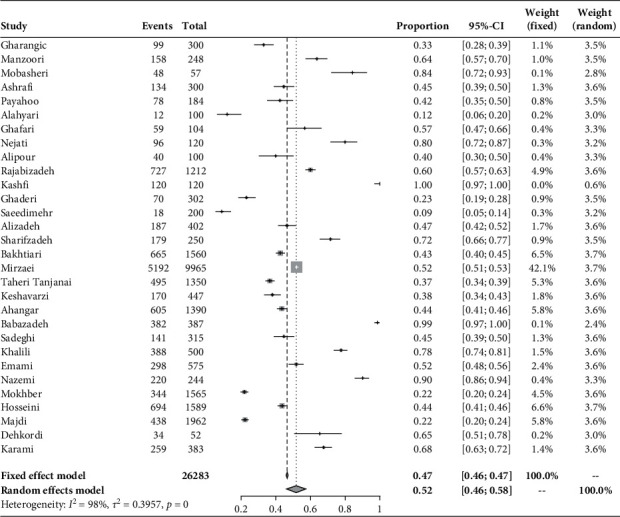
Forest plot of the selected studies.

It can be found in Figure 2 that the synthesized prevalence of depression among the elderly people in Iran for the random-effects model was 52% (95% CI: 46–58). The Q-test for pooled estimates was statistically significant at the level of 0.001 (*Q* = 1890.68, d*f* = 29, *p* < 0.001, *I*2 = 98%) representing noticeable between-study heterogeneity.

**Figure 3:**
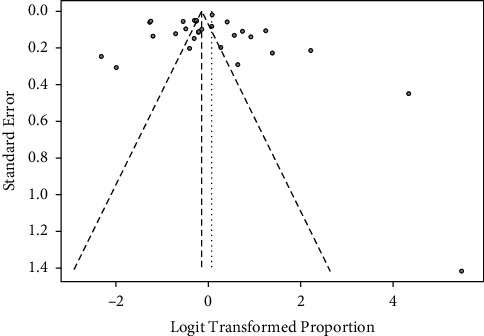
Funnel plot of the selected studies.

The publication bias was also evaluated by visualizing the funnel plot for analysis. The funnel plot (Figure 3) depicts how heterogeneous the effect sizes of the elected studies are. The *p* value associated with Egger's test is 0.748, indicating symmetry of the funnel plot.

## 4. Discussion

According to the results of this study, the prevalence of depression in the Iranian elderly was 52%. In a similar study, the prevalence of depression in the Iranian elderly was 43 percent, according to a review of studies conducted between 2001 and 2015 [23]. Comparison of the results of the present study and the mentioned study [23] indicates that the prevalence of depression in the Iranian elderly has increased over time. Also, another study with a review of 83 studies in this field reported that 27 percent of the elderly suffer from depression [51], with the prevalence of depression in this study being less than the prevalence of depression in the Iranian elderly. Possible reasons for the difference in the prevalence of depression in these studies may be the structures intended by governments and the culture of societies in relation to elderly people and their needs. In some countries, especially developed countries, there is a more favourable formalized mental healthcare system for the elderly, which can affect the prevention and promotion of their mental health, especially depression screening. Also, increasing rates of disease and medicine consumption in developing countries such as Iran may increase the experience of depression in the elderly.

In the present study, the lowest prevalence of depression was in the study by Saeedi et al. [37] and the highest was in the study by Kashafi et al. [35]. Possible reasons for the differences in results may be the differences in the place of study and the different cultural contexts and tools used. Saeedi et al.'s study [37] was conducted in Ahvaz, and another study was conducted in Shiraz. In the study of Saeedi et al. [37], the Geriatric Depression Scale (GDS) was used and, in the latter, the Beck Depression Inventory was used. Also, in the study by Saeedi et al., the elderly surveyed were retirees of the Ahwaz oil industry, while in the other study, not all of the subjects were retired from a particular organization. Not all individuals in Kashafi et al.'s study [35] (unlike Saeedi et al.'s study) did not use retirement benefits and thus may experience more economic and social problems and have less access to health care and support. This may be the cause of the difference in the prevalence of depression in the two mentioned studies.

### 4.1. Limitations

Despite the strengths of the study, restrictions such as not searching studies in languages other than Persian and English prevented access to all studies in this field.

## 5. Conclusion

According to the results of this study, depression in the Iranian elderly was moderate to high. Therefore, more exact assessment in terms of depression screening in elderly people seems necessary. According to the World Health Organization's motto of prevention prior to treatment, health managers can use the results of this study and emphasize to screen for depression in nursing homes as well as in rural health homes. Coherent and systematic programs, including psychosocial empowerment counselling for the elderly and workshops for their families, are also needed. Researchers can also use the results of this study for future research.

## Figures and Tables

**Table 1:** Characteristics of the included studies (*N* = 30).

Author (year) (reference)	Study type	Place of study	Instrument	Sample, *n* (%)	Average of age, mean (SD)	Prevalence of depression, *n* (%)	Quality of study
Gharangic (2010) [27]	Cross-sectional	Bandar torkaman	GDS-15	Male: 155 (51.7) Female: 145 (48.3)	68 (6.7)	Normal: 201 (67) Mild: 60 (20) Moderate: 30 (10) Severe: 9 (3) Total depressed: 33%	7
Manzoori (2007) [28]	Cross-sectional	Isfahan	GDS-15	Male: 125 (50.4) Female: 123 (49.6)	Not reported	Normal: 90 (36.3) Moderate: 101 (40.7) Severe: 57 (23) Total depressed: 63.7%	7
Mobasheri (2008) [29]	Descriptive-analytical	Shahrekord	BDI-21	57	68.7 (16.1)	Normal: 9 (15.8) Mild: 36 (64.9) Moderate: 9 (15.8) Severe: 2 (3.5) Total depressed: 84.2%	7
Ashrafi (2017) [30]	Cross-sectional	Salmas	GDS-15	Male: 137 (45) Female: 163 (55)	68.74 (6.32)	Normal: 166 (55.3) Mild: 84 (28) Moderate: 31 (10.33) Severe: 19 (6.3) Total depressed: 44.7%	7
Payahoo (2012) [31]	Descriptive-analytical	Tabriz	GDS	Male: 97 (52.7) Female: 87 (47.3)	69.4 (7.9)	Normal: 106 (57.6) Moderate: 52 (28.3) Severe: 26 (14.1) Total depressed: 42.4%	7
Alahyari (2014) [32]	Descriptive-analytical	Tehran	Beck	100	Not reported	Total depressed: 12%	7
Ghafari (2009) [22]	Descriptive-analytical	Tehran	DASS-21	Male: 25 (24) Female: 79 (76)	64.22 (4.57)	Normal: 45 (43.2) Mild: 16 (15.4) Moderate: 30 (28.9) Severe: 8 (7.7) Very severe: 5 (4.8) Total depressed: 56.8%	7
Nejati (2006) [33]	Cross-sectional, comparative	Tehran	GDS	120	Not reported	Total depressed: 79.8%	7
Alipour et al. (2009) [12]	Comparative-analytical	Tehran	HADS	Male: 70 (70) Female: 30 (30)	76.97	Total depressed: 40%	7
Rajabizadeh (2002) [34]	Cross-sectional	Kerman	Beck	1212	Not reported	Normal: 259 (21.4) Borderline: 225 (18.6) Depressed: 728 (60) Total depressed: 60%	7
Kashfi (2011) [35]	Cross sectional	Shiraz	Beck	Male: 20 (16.7) Female: 100 (83.3)	70	Mild: 78 (65) Moderate: 31 (25.8) Severe: 11 (9.2) Total depressed: 100%	7
Ghaderi (2010) [36]	Descriptive, cross-sectional	Bookan	GDS	302	70.69	Total depressed: 23.3%	7
Saeedi (2009) [37]	Cross-sectional	Ahvaz	GDS	Male: 66 (33) Female: 134 (67)	71 (8)	Total depressed: 9%	8
Alizadeh (2012) [21]	Descriptive-analytical	Tehran	Kessler	402	Not reported	Normal: 189 (47.1) Moderate: 187 (46.5)	7
Sharifzadeh (2007) [38]	Descriptive-analytical	Birjand	Elderly health master plan	250	71 (7.8)	Normal: 71 (28.4) Mild: 171 (68.4) Severe: 8 (3.2) Total depressed: 71.6%	7
Bakhtiari (2018) [18]	Cross-sectional	Amirkola	GDS	Male: 861 (55) Female: 699 (45)	69.3 (7.4)	Normal: 895 (57.4) Mild: 420 (26.9) Moderate: 169 (10.8) Severe: 76 (4.9) Total depressed: 42.6%	7
Mirzaei (2015) [15]	Prospective	Yazd	DASS	9965	Not reported	Normal: 473 (47.9) Mild: 420 (26.9) Moderate: 169 (10.8) Severe: 76 (4.9) Total depressed: 52.1%	7
Taheri tanjanai (2016) [39]	Cross-sectional	Tehran	GDS-15	Male: 642 (47.5) Female: 708 (52.5)	69 (7)	Total depressed: 36.7%	8
Keshavarzi (2015) [11]	Cross-sectional	Shiraz	GDS	Male: 125 (27.9) Female: 322 (72.1)	65.99 (7.89)	Total depressed: 38.1%	7
Ahangar (2017) [40]	Cross-sectional	Amirkola	GDS-15	Male: 763 (54.9) Female: 627 (45.1)	68.87 (7.23)	Total depressed: 43.5%	7
Babazadeh (2016) [41]	Cross-sectional	Khoy	DASS-21	Male: 178 (45.4) Female: 209 (54.6)	68.22	Mild: 167 (43.6) Moderate: 71 (18.5) Severe: 134 (35.0) Very severe: 6 (1.6) Total depressed: 98.7%	7
Sadeghi (2017) [42]	Cross-sectional analytical	Shahrood	GDS-15	Male: 146 (46) Female: 169 (54)	Not reported	Mild: 96 (30.4) Severe: 45 (14.3) Total depressed: 44.7%	7
Khalili (2016) [43]	Descriptive, cross-sectional	Kashan	GDS-15	Male: 290 (58) Female: 210 (42)	72.07 (9.03)	Normal: 112 (22.4) Mild: 239 (47.8) Moderate: 120 (24) Severe: 29 (5.8) Total depressed: 77.6%	7
Emami (2017) [44]	Cross-sectional	Tehran	BDI-13	Male: 227 (32.48) Female: 348 (60.52)	69.66 (7.91)	Normal: 271 (48.13) Mild: 98 (17.41) Moderate: 144 (25.58) Severe: 50 (8.88) Total depressed: 51.87%	8
Nazemi (2013) [45]	Descriptive-analytical	Tehran	GDS-15	Male: 130 (53.3) Female: 114 (46.7)	75.8 (8.7)	Normal: 24 (9.8) Mild: (50) Moderate: (29.5) Severe: (10.7) Total depressed: 90.2%	8
Mokhber (2011) [46]	Analytical	Razavi khorasan	GDS	Male: 720 (46) Female: 845 (54)	70.14 (7.57)	Nondepressed: 1165 (78) Depressed: 330 (22)	7
Hosseini (2018) [47]	Cross-sectional	Babol	GDS-15	Male: 864 (54.4) Female: 725 (45.6)	69.38 (7.44)	Normal: 895 (56) Mild: 436 (27) Moderate: 176 (11) Severe: 82 (5) Total depressed: 43.67%	7
Majdi (2011) [48]	A population-based study	Razavi khorasan	GDS	Male: 917 (46.4) Female: 1045 (52.9)	71.14 (7.78)	Nondepressed: 1435 (72.2) Depressed: 440 (22.3)	7
Dehkordi (2014) [49]	Descriptive-analytical	Shahrekord	GDS-15	52	72.9 (6.5)	Normal: 14 (34.2) Mild: 16 (39.1) Moderate: 7 (17) Severe: 4 (9.7) Total depressed: 65.8%	7
Karami (2014) [50]	Descriptive-analytical	Kermanshah	Beck	Male: 223 (58.2) Female: 160 (41.8)	70.11 (5.7)	Normal: (32.4) Mild: (18.5) Moderate: (40.2) Severe: (5.7) Very severe: (3.1) Total depressed: 67.6%	7

## Data Availability

The data used in the study are available from the corresponding author upon request via e-mail.
